# Naturally occurring antibodies isolated from PD patients inhibit synuclein seeding in vitro and recognize Lewy pathology

**DOI:** 10.1007/s00401-019-01974-5

**Published:** 2019-02-25

**Authors:** Xinyi Li, Wouter Koudstaal, Lauren Fletcher, Martha Costa, Margot van Winsen, Berdien Siregar, Hanna Inganäs, Julie Kim, Elissa Keogh, Jeremy Macedo, Trevin Holland, Stuart Perry, Frederique Bard, Jeroen J. Hoozemans, Jaap Goudsmit, Adrian Apetri, Gabriel Pascual

**Affiliations:** 1 Janssen Prevention Center, Janssen Pharmaceutical Companies of Johnson & Johnson, 3210 Merryfield Row, San Diego, CA 92121 USA; 2 Janssen Prevention Center, Janssen Pharmaceutical Companies of Johnson & Johnson, Archimedesweg 6, 2333 CN Leiden, The Netherlands; 3 Department of Pathology, Amsterdam Neuroscience, VU University Medical Center, De Boelelaan 1117, 1081 HV Amsterdam, The Netherlands; 4 Department of Epidemiology, Harvard T.H. Chan School of Public Health, 677 Huntington Avenue, Boston, MA 02115 USA; 5 Department of Neurology, Amsterdam Neuroscience, Academic Medical Center, Meidreefberg 9, 1105 AZ Amsterdam, The Netherlands; 6 Present Address: Lucidity Biomedical Consulting, Calle Emir 11, 18006 Granada, Spain

**Keywords:** Parkinson’s disease, Alpha-synuclein protein, Memory B cell, Monoclonal antibody, Lewy bodies, Lewy neurites

## Abstract

Deposition of α-synuclein into Lewy bodies and Lewy neurites is the hallmark of Parkinson’s disease (PD). It is hypothesized that α-synuclein pathology spreads by a “prion-like” mechanism (i.e., by seeded aggregation or templated misfolding). Therefore, various extracellular α-synuclein conformers and/or posttranslational modifications may serve as biomarkers of disease or potential targets for novel interventions. To explore whether the antibody repertoires of PD patients contain anti-α-synuclein antibodies that can potentially be used as markers or immunotherapy, we interrogated peripheral IgG^+^ memory B cells from PD patients for reactivity to α-synuclein. In total, ten somatically mutated antibodies were recovered, suggesting the presence of an ongoing antigen-driven immune response. The three antibodies that had the highest affinity to recombinant full-length α-synuclein, aSyn-323.1, aSyn-336.1 and aSyn-338.1, were characterized further and shown to recognize epitopes in the C terminus of α-synuclein with binding affinities between 0.3 and 2.8 μM. Furthermore, all three antibodies were able to neutralize the “seeding” of intracellular synuclein aggregates in an in vitro α-synuclein seeding assay. Finally, differential reactivities were observed for all three human anti-α-synuclein antibodies across tissue treatment conditions by immunohistochemistry. Our results suggest that the memory B-cell repertoire of PD patients might represent a potential source of biomarkers and therapies.

**Electronic supplementary material:**

The online version of this article (10.1007/s00401-019-01974-5) contains supplementary material, which is available to authorized users.

## Introduction

Lewy bodies (LB) and Lewy neurites (LN), the neuropathological hallmarks of Parkinson’s disease (PD), are abnormal protein depositions generated by the misfolding and aggregation of α-synuclein, which is a natively disordered, 14 kD protein mostly localized to presynaptic terminals involved in vesicular transport. Combined with Dementia with Lewy bodies (DLB), PD represents the second most common dementia among elderly people. Multiple system atrophy (MSA) and some lysosomal-storage diseases, such as Gaucher’s disease [34] also exhibit α-synuclein aggregation. Moreover, non-amyloid component (NAC) fragment of α-synuclein was found in amyloid-β (Aβ) plaques in Alzheimer’s disease (AD), and 50% of AD cases show Lewy Body pathology [10]. Moreover, α-synuclein has been suggested to regulate aggregation of Aβ [3] and tau [18], two proteins associated with neuropathological hallmarks of AD. Point mutations (A30P, E46 K, H50Q, G51D, A53E and A53T) of α-synuclein protein and increased dosage of *SNCA*, the gene encoding α-synuclein, are associated with familial forms of PD [23, 44]. Through genome-wide association studies (GWAS), *SNCA* was identified as one of the most important genetic risk factors for idiopathic PD [11]. Moreover, GWAS identified alleles of major histocompatibility complex (MHC) that are associated with PD. MHC class II, DRB5*01, DRB1*15:01 and non-coding SNPs enhancing MHC class II expression are positively associated with PD [19, 21, 43]. T cells from PD patients recognize α-synuclein peptides, particularly peptides 31–45, 32–46 and 116–140 (phosphorylated S129 region), and produce markedly higher IL-5 and IFNγ responses compared to age-matched controls [38]. Recently, Sommer et al. reported increased IL-17 producing T cells in brains and blood of PD patients [36]. Using human-induced pluripotent stem cells (hiPSC)-derived neurons and autologous co-culture with T cells, they demonstrated IL-17-mediated neuron death in PD patient-derived cell co-cultures [36]. These findings strongly support a critical role of adaptive immunity in PD.

Analysis of LB pathology showed a progressive spreading of α-synuclein aggregates with disease progression or clinical progression of PD, suggesting that spreading of extracellular α-synuclein aggregates is the driver of disease pathology [7]. The neo-epitopes associated with aggregated synuclein or elevated extracellular synuclein concentration likely activate the host immune system and result in T-cell and B-cell activation and production of auto-antibodies against synuclein protein. Indeed, increased levels of auto-antibodies binding to recombinant synuclein have been reported in early PD patients [16, 17, 22, 35] and in populations carrying genetic risk factors [1, 29], which might be in an asymptomatic early stage of PD. Interestingly, however, several studies observed no increase in auto-antibodies against synuclein in PD patients [1, 5, 20, 29].

In a previous study, we have shown that the immune repertoires of both healthy controls and patients with AD contain naturally occurring antibodies against tau [2, 30, 41]. In the present study, we interrogated IgG^+^ memory B cells from PD patients for antibodies against α-synuclein and recovered 10 mAbs, a subset of which showed functional activity in an in vitro synuclein seeding assay and recognized pathological LB and LN in PD tissue.

## Materials and methods

### Human PBMC preparation

Whole blood (100 ml) from 25 clinically diagnosed PD patients (aged 50–65) was purchased from Sanguine Biosciences. Peripheral blood mononuclear cells (PBMCs) were isolated and cryopreserved as previously described [30]. Briefly, the cells were isolated on Ficoll-Paque Plus (GE healthcare) and cryopreserved in 90% FBS and 10% DMSO.

### Peptide synthesis

To screen and clone naturally occurring human mAbs to α-synuclein protein, a panel of 7 peptides covering the center region and C-terminus of α-synuclein (amino acids 61–140) were designed and synthesized (Table S1). The peptides included phosphorylation at Ser-129 and Ser-87 and truncation at amino acid positions 110 and 120. The peptides were synthesized by solid-phase chemistry and their purity was confirmed to be higher than 95% by LC–MS (New England Peptide, Inc. and Eton Bioscience, Inc.). Biotinylated peptides were synthesized by connecting biotin moieties via a LC linker to either the N- or C- terminus of the peptides.

### Synuclein constructs for protein production and seeding assay

Full-length (140 amino acids) human α-synuclein gene *SNCA* was synthesized at Genewiz Inc and cloned in an pUC57 entry vector. The Avi-α-synuclein sequence was codon optimized for bacterial expression. Xbal and NotI sites were introduced by PCR using Phusion High Fidelity PCR Master Mix (Thermo Fisher); the PCR products were double digested (NEB), gel purified, and ligated into pET28 vector following the manufacturer’s protocol to generate His–Thrombin–Avi-tagged full-length protein. Human *SNCA* was PCR amplified from a pUC57 vector (Genewiz Inc) with primers (Eton Bioscience) overlapping with SNCA and pcDNA2004 vector and 3′ primers with sequences encoding either -V5 or -HA tag. The fragments were subsequently gel purified and assembled into the vector using a Gibson Assembly Cloning Kit (NEB) following the manufacturer’s protocol. The products were transformed into DH5α competent cells (Thermo Fisher) and the plasmids were confirmed by sequencing. To generate a negative control for the seeding assay, an α-synuclein recombinant version lacking amino acids 60–93 was generated. Primers were designed to amplify two fragments (1–59 and 94–140) of α-synuclein. Q5 high fidelity master mix (NEB) was used for PCR (12 cycles) using pcDNA2004 vector with inserts of SNCA-V5 or SNCA-HA as template. The resulting PCR product was treated with DpnI (NEB) and then transformed into DH5α competent cells (Thermo Fisher), and colonies were sequenced to confirm the correct deletion (amino acid 61–92 deletion). Finally, the expression of the α-synuclein with the different tags was confirmed in HEK293 cells by Western blot of cell lysate samples.

### Purification and biotinylation of recombinant synuclein

Full-length wild-type α-synuclein, with a C-terminal Avi-tag, thrombin cleavage site and His-tag, was produced in *E.coli* BL21 (DE3) (Thermo Fisher) cells in a 10-L wave bag. 3 h after induction with IPTG (Sigma-Aldrich), the cells were harvested, and the pellets were stored at − 80 °C. The pellets were re-suspended and thawed in lysis buffer (BugBuster Master mix, Millipore) with protease inhibitor cocktail (cOmplete Ultra EDTA free, Roche). The suspension was centrifuged, and the supernatant was heated for 1 h at 60 °C; then centrifuged at 5250 x g at 4 °C for 30  min. The supernatant was buffer exchanged to 50 mM Bicine pH 8.3. Size-exclusion chromatography with multi-angle static light scattering (SEC–MALS) analysis was used to estimate the total amount of α-synuclein. The required amounts of BirA enzyme, biotin, ATP and magnesium acetate were added for biotinylation overnight per manufacturer instructions (BirA biotin–protein ligase bulk reaction kit, Avidity LLC). Biotinylation was confirmed by SEC–MALS analysis of biotin–synuclein binding to streptavidin-PE. The material was applied to a His-tag resin (Roche Complete His-tag purification Resin), washed three times to remove impurities and the α-synuclein was eluted by thrombin during overnight incubation in 200 mM Tris-HCl, 1.5 M NaCl, 25 mM CaCl2, pH 8.4 and purified on a Superdex 75 SEC column. The protein was quantified by absorption measurements at 280 nm using an extinction coefficient of 0.59 ml mg^−1^ cm^−1^. The high purity of the biotinylated α-synuclein was confirmed by SDS-PAGE and analytical SEC (Fig. S1a and b). In addition, purified and biotinylated α-synuclein was mixed with streptavidin-PE and analyzed by SEC–MALS, showing that all α-synuclein was indeed biotinylated (Fig. S1c). The reactivity of biotinylated α-synuclein was assessed by ELISA using a streptavidin-coated plate (described below). The protein is fully reactive to antibodies Syn303 (Biolegend) and C20 (Santa Cruz Biotechnology), which recognize N-terminal (amino acids 1–5) and C-terminal (amino acids 120–140) of synuclein, respectively.

### Generation of α-synuclein baits and single cell sorting of bait-specific memory B cells

Synuclein peptide and protein baits were prepared by mixing biotinylated peptides or proteins with streptavidin-APC or streptavidin-PE (Thermo Fisher). The majority of peptides and free biotin (a negative control) were prepared at a 1:9 ratio (SA:peptide), incubated for 15 min on ice and passed over a BioSpin 30 column (Biorad) to remove free peptide. The full-length protein and the aggregation prone C-terminally biotinylated peptide 61–95 were prepared at a 1:4 ratio and were used without column clean-up. Each tetramer was used at a final concentration of 36 nM, based on the streptavidin concentration (Table S1). Identification of antigen-specific, memory B cells was performed as previously described [30]. Briefly, PBMCs from 4–6 PD donors were thawed and rested overnight in complete RPMI media (RPMI with 10% FBS and 1% pen/strep) at 37 °C. The B cells were enriched by positive selection with CD22+ magnetic beads (Miltenyi Biotec). Cells were labeled at a final concentration of 20 million per ml in FACS buffer [Tris-buffered saline (TBS) at pH 7.4, with 2 mM EDTA and 0.25% bovine serum albumin (BSA), Fraction V] with the extracellular markers IgG-FITC, CD19-PerCPCy5.5, and CD27-PECy7 (all from BD Biosciences) and the dual-labeled protein/peptide tetramers. To determine nonspecific binding of the tetramers, antibody-labeled cells were incubated with the biotin tetramers, used at the concentration of the pool of peptides (for 8 peptides = 288 nM). The cells and peptides were incubated for 1 h at 4 °C with gentle mixing. After washing, the cells were resuspended at 20 x 10^6^/ml in FACS buffer. The live/dead marker DAPI (Thermo Fisher) was added before the cells were sorted on a Beckman Coulter MoFlo XDP. The gates were set using the negative control and the CD19+, IgG+, CD27^hi^, and antigen double-positive live cells were collected by single-cell sorting directly into PCR plates containing cold RT-PCR reaction buffer and RNaseOUT (Thermofisher). Plates were centrifuged briefly and stored at − 80 °C.

### Recovery of heavy- and light-chain antibody genes from memory B cells

Heavy- and light-chain (HC/LC) antibody variable regions were recovered using a two-step PCR as described previously [30]. Briefly, cDNA of variable chain fragments was synthesized using Superscript III First Strand Synthesis Kit (Thermo Fisher), then amplified by nested PCR using pooled forward primers for leader sequence and reverse primers specific to Cγ, Cκ, and Cλ. The step II PCR fragments were subsequently linked via overlap extension PCR followed by digestion of the product with XbaI and XhoI (New England Biolabs). The digested product was subsequently cloned into a dual-CMV-based human IgG1 mammalian expression vector.

### Recombinant IgG expression

Cloned mAbs were transiently transfected into Expi293™ cells (Thermo Fisher) and media were harvested by centrifugation at 72 h post transfection. The IgG was purified from the culture media by Protein A affinity chromatography as previously described [30]. The IgGs were quantified by UV–VIS absorption at 280 nm, and the quality of IgGs was examined by SEC–MALS to assess the monomer content and the amount of aggregates and SDS-PAGE under reducing and non-reducing conditions to assess purity. All IgGs employed in our studies were more than 98% monomeric.

### Synuclein pre-formed fibrils (PFF)

Monomeric, full-length α-synuclein, generated as described above, was aggregated by incubation for 5–6 days at 37 °C in a rotator in the presence of small Teflon beads (1/16”). The samples were centrifuged for 15 min at 20,000 g to separate monomers/oligomers and aggregates. The pellet was stored at − 80 °C to be used as bait and the supernatant was injected on SEC–MALS to quantify the α-synuclein content in the pellets.

### Synuclein seeding assay

Antibody-conjugated beads were prepared following Schrum et al. [33]. Briefly, carboxyl groups on CML Latex Beads (Sigma) were activated with EDAC (50 mg/ml) dissolved in MES coupling buffer (50 mM MES pH 6.0, 1 mM EDTA). Mouse monoclonal anti-V5 antibody (Sigma) in phosphate-buffered saline (PBS) was added to the beads with shaking for 3–4 h, and then washed for later use. HEK293 cells (ATCC, less than 30 passages) were plated (20,000 per well) in a 96-well plate (Costar) in DMEM high-glucose media (Cellgro) supplemented with 10% FBS (Gibco), 1% penicillin, 1% streptomycin and 1% l-glutamine (Hyclone) and left overnight at 37 °C in 8% CO_2_. The cells were then transfected using FuGENE HD (Promega). Briefly, 50 ng of Syn-HA and 50 ng Syn-V5 plasmids (or Syn-HA with negative control plasmid pcDNA-SNCA-Del61-92-V5) and 0.3 μl of FuGENE were mixed, incubated for 10 min, and then 10 μl of complexes were incubated with cells at 37 °C for 24 h.

Synuclein aggregates were thawed at room temperature for 15 min, followed by vortexing and dilution to 1 mg/ml. 4 μg of aggregates and 200 μg of anti-synuclein antibodies were mixed to a final volume of 50 μl in PBS (Gibco), incubated for 2 h at 37 °C with shaking and diluted with 350 μl of medium. Finally, 100 μl of the mixture was added to each of four wells and incubated for 72 h. Control antibodies used included mouse Syn211 which prevented synuclein PFF uptake and cell-to-cell transmission of pathology [39] (positive control, ThermoFisher), mouse anti-FLAG M2 (isotype control, Sigma) and human anti-RSV antibody (human isotype control). Cells were detached by adding 50 μl of 0.25% Trypsin-EDTA PBS (Gibco). Then, 150 μl of media was added, and the cells were collected by centrifugation. The cells were lysed in 100 μl of ice-cold Lysis Buffer [1% Triton-X (Sigma) in TBS (Quality Biological)] supplemented with protease inhibitors (Roche) on ice. The lysates were centrifuged to remove cell debris (3000 x g, 5 min, at 4 °C) and 80 μl of supernatant was transferred into a cold 96-well round bottom plate. 150,000 beads in 10 μl (TBS + 1% Triton X-100 and 1X protease inhibitors) were added to each well and incubated overnight at 4 °C with shaking at 750 rpm on a Microplate Genie (USA Scientific). The next day, the beads were centrifuged and washed twice in 200 μl ice-cold Post-IP Buffer (0.2% TritonX-100 in Lysis Buffer).

The capture beads were washed two times in ice-cold FCM staining buffer (BD) by centrifugation. Mouse anti-HA-SureLight APC antibody (Columbia Biosciences) was added to each sample and incubated 40–60 min at 4 °C. The samples were washed three times in FCM Staining Buffer, resuspended in 200 μl FCM staining buffer, and flow cytometry analysis was performed using the MACSQuant (Miltenyi Biotec). The percent positive signal was calculated as the number of APC-positive particles divided by the total number of particles and normalized against that of samples incubated with seeds only (no antibody).

### ELISA

Pierce streptavidin-coated 96-well plates or Costar high-binding plates were coated with the individual biotinylated synuclein peptides (400 nM final) or control (bovine actin, 1 μg/ml) diluted in TBS overnight at 4 °C, respectively. The IgG concentration of the antibodies was determined by Octet with Protein A biosensors using a Protein A calibrator set (ForteBio). The anti-synuclein IgGs, diluted to 10 µg/ml in TBS-T (TBS containing 0.05% Tween 20 and 0.25% BSA), were added to the blocked wells in duplicate and incubated at room temperature for 2 h. After washing, goat-anti human IgG Fab-HRP (1:2000) or goat anti-mouse-HRP (1:4000, Jackson Labs) was added and incubated for 1 h. Plates were washed five times with TBS-T and developed with 100 μl SureBlue Reserve TMB Microwell Peroxidase Substrate (KPL). The reaction was stopped by the addition of 100 μl of TMB stop solution (KPL), and the absorbance at 450 nm was measured using a Tecan M1000 plate reader. Antigen-specific binding was defined as an OD450 greater than 0.5 and at least threefold above the secondary antibody alone. To confirm these results, the antibodies that met the criteria for antigen specificity were serially diluted fivefold in TBST from a starting concentration of 10 μg/ml and retested against the antigen for which they demonstrated reactivity.

### Qualitative association and dissociation measurements by Octet biolayer interferometry

The relative binding of the antibodies to full-length synuclein was assessed by biolayer interferometry measurements (Octet Red 384, ForteBio) [8]. Biotinylated synuclein protein was immobilized on Streptavidin (SA) Dip and Read biosensors for kinetics (ForteBio). Real-time binding curves were measured by applying the sensor in a solution containing 100-nM antibody. To induce dissociation, the biosensor containing the antibody–synuclein complex was immersed in assay buffer without antibody. The immobilization of synuclein to sensors, the association and the dissociation steps, were followed in different ionic strength buffers containing 10% FortéBio kinetics buffer as assay buffer. The relative association and dissociation kinetic curves were compared to qualitatively assess the efficiency of antibody binding to peptides encompassing different synuclein epitopes.

### Affinity measurements by Isothermal Titration Calorimetry (ITC)

The affinities of antibodies for synuclein peptides were determined in solution on a MicroCal Auto-iTC200 system (Malvern). Synuclein peptides at 40 μM were titrated in 20 steps of 2 μl per step, in identical buffers containing 200 μM aSyn-323.1, aSyn-336.1 and aSyn-338.1, respectively. The thermodynamic parameters and the equilibrium dissociation constants, Kd, were determined upon fitting the ITC data to a model assuming a single set of binding sites corresponding to an antibody:synuclein = 1:2 binding model.

### Immunohistochemistry on post-mortem human brain tissue

Post-mortem human brain tissue was obtained from the VU University Medical Center. Sections (5-µm thick) from formalin-fixed paraffin-embedded PD brain tissue (mesencephalon containing the substantia nigra pars compacta) were mounted on coated glass slides (Menzel gläser superfrost plus, VWR international) and dried overnight at 37 °C. Slides were deparaffinized in xylene and rehydrated through descending alcohol concentrations. Endogenous peroxidase activity was blocked by incubating the slides for 30 min in PBS (pH 7.4) containing 0.3% H_2_O_2_. Before immunodetection, sections were untreated, treated with formic acid for 30 min at RT or autoclaved for 20 min in 10 mM citric acid pH6.0. Between incubation steps, sections were rinsed in PBS. All antibodies were diluted in antibody diluent (Immunologic) and incubated overnight at RT. Human anti-α-synuclein antibodies aSyn-323.1, aSyn-336.1 and aSyn-338.1 were used at a concentration of 0.5 μg/ml. Mouse anti-alpha-synuclein antibody LB509 (Thermofisher) was used at a concentration of 1.25 μg/ml. Primary antibodies were detected with goat-anti-human-HRP (dilution 1:250, 60 min at RT, Santa Cruz) or goat-anti-mouse/rabbit-HRP (ready-to-use, 30 min at RT, EnVision Dako). To visualize the staining 3,3′-diaminobenzidine (DAB; Dako) was used. Slides were counterstained with haematoxylin, dehydrated and mounted with Quick-D mounting medium (Klinipath). Different antibodies were assessed in serial tissue sections.

## Results

Blood samples from 25 PD patients were screened for the presence of memory B cells reactive to α-synuclein using our previously described BSelex method [30]. Certain posttranslational modifications (PTMs), alternative splicing, and truncations are associated with synuclein aggregation and neurotoxicity [6]. For example, phosphorylation of Ser129 and Ser87 is associated with LB pathology of PD [13, 28], and C-terminal truncations leading to fragments encompassing amino acids 1–110 and 1–120 are conducive of α-synuclein aggregation, which are toxic in vivo [24, 40]. This propensity to aggregate is conferred by the NAC region (amino acid 61–95), and the aggregate species generated are neurocytotoxic [12]. Therefore, in addition to recombinant full-length α-synuclein protein, we used a panel of peptides, including these common PTMs and critical fragments as antigen baits for the recovery of α-synuclein antibodies from PD patient samples (Table S1).

**Fig. 1:**
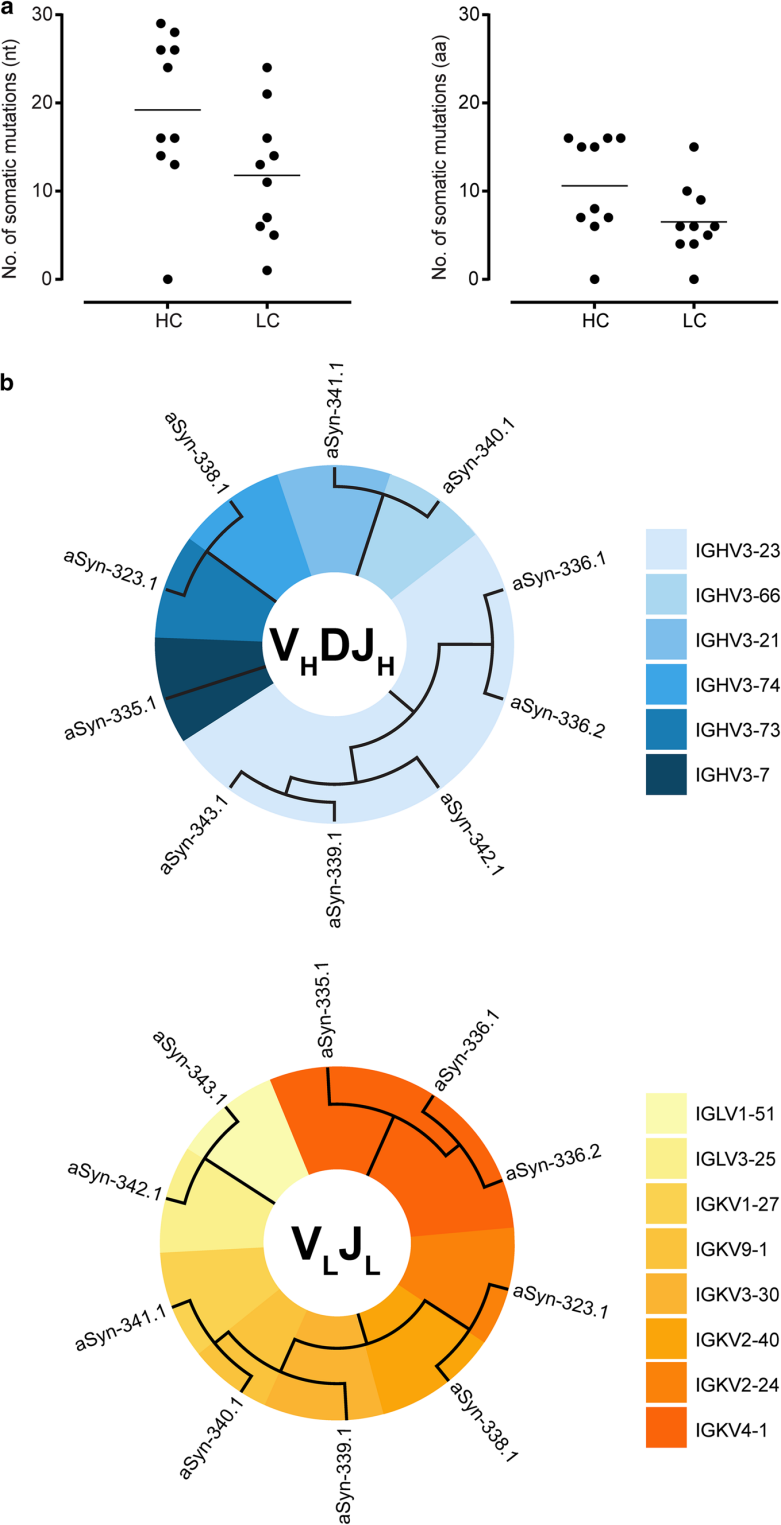
Sequence analyses of recovered anti-α-synuclein antibodies. **a** Number of somatic mutations in V_H_ and V_L_ genes of 10 antibodies recovered from IgG^+^ memory B cells with reactivity to α-synuclein. The closest germline and mutations were determined using IgBlast and IMGT databases. The horizontal lines indicate medians **b** Phylogenetic analysis of recovered antibody heavy- and light-chain variable regions was performed using the neighbor-joining algorithm (Jukes cantor model) and illustrated as circular trees

**Fig. 2:**
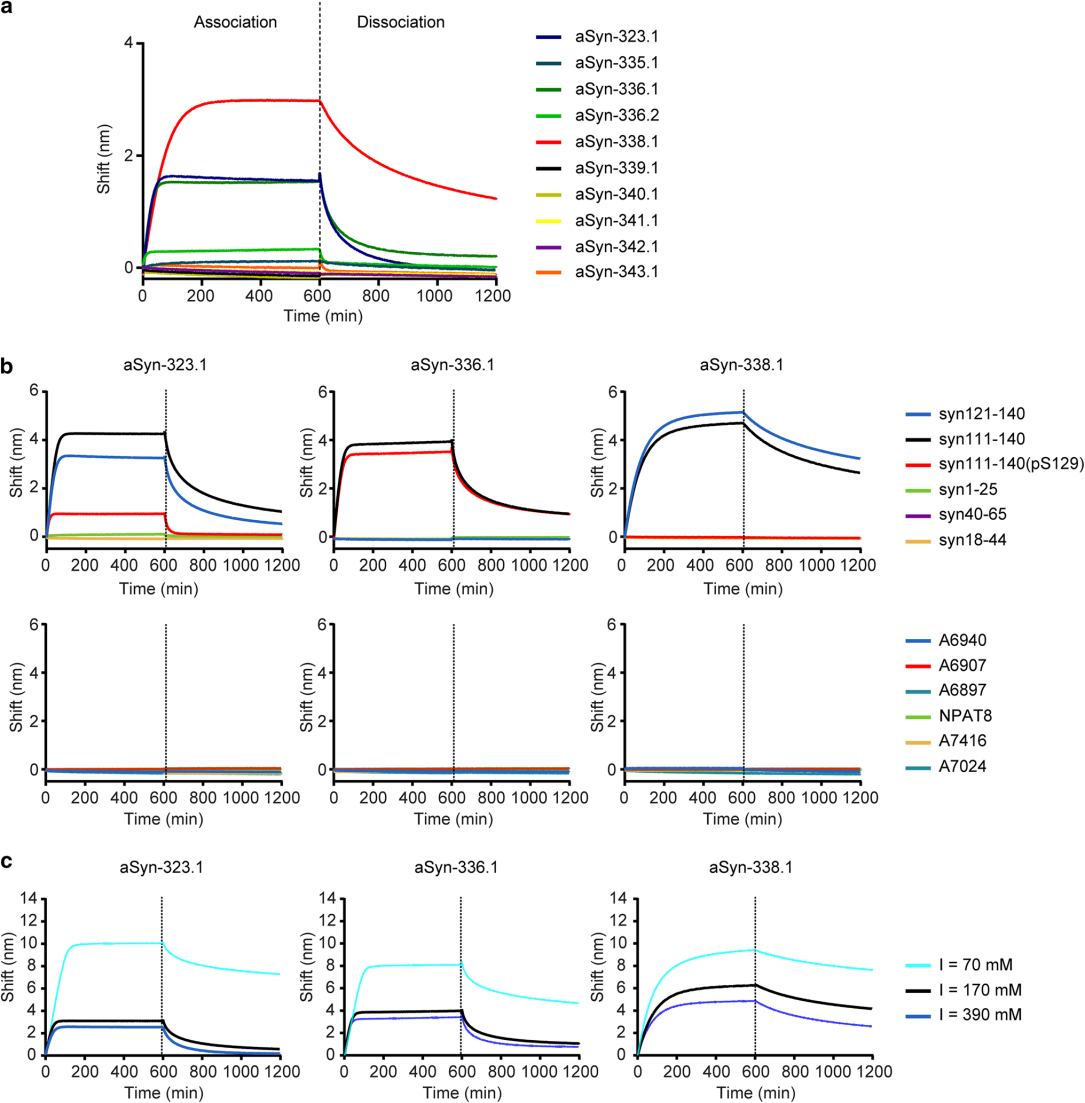
Binding to full-length synuclein protein, epitope mapping and nature of interactions of anti-α-synuclein antibodies. **a** Association (0–600 s) and dissociation (600–1200 s) profiles for recovered aSyn monoclonal antibodies to biotinylated full-length synuclein as determined by Octet biolayer interferometry. **b** Association (0–600 s) and dissociation (600–1200 s) kinetics for the binding of aSyn-323.1, aSyn-336.1, and aSyn-338.1 to peptides encompassing different amino acid sequences of the synuclein protein (top row) and off-target binding as assessed against a panel of different tau peptides (bottom row) as determined by Octet biolayer interferometry. The sequence of each peptide is shown in Table S1. **c** Association (0–600 s) and dissociation (600–1200) kinetics for the binding of aSyn-323.1 to syn121–140 (left), aSyn-336.1 to syn111–140 (center) and aSyn-338.1 to syn121–140 (right) were determined at different ionic strengths. The significant effect of ionic strength on antibody binding to synuclein peptides indicates that the interactions are stabilized by electrostatic forces

In total, ten unique antibodies were recovered. Analysis of heavy- and light-chain germline usage revealed considerable numbers of variable region somatic mutations (Fig. 1a and b), which, in combination with the fact that the antibodies were retrieved from the IgG compartment and are, thus, isotype switched, suggests the presence of ongoing antigen-driven responses. Next, binding of the antibodies to full-length recombinant α-synuclein was assessed by Octet Biolayer Interferometry (Fig. 2a). The three antibodies that showed the strongest binding—aSyn-323.1, aSyn-336.1 and aSyn-338.1—were characterized further. Peptide epitope mapping revealed that while all three antibodies bound to a peptide covering α-synuclein residues 111–140, only aSyn-323.1 and aSyn-338.1 bound to a peptide encompassing residues 121–140 (Fig. 2b). Antibody aSyn-336.1 failed to react with the peptide spanning region 121–140, indicating that its epitope is located between residues 111 and 121, while the other two antibodies bind more C-terminally, between residues 121 and 140. Furthermore, phosphorylation of Ser129 [as present in peptide syn111-140(pS129)] greatly diminished binding of both aSyn-323.1 and aSyn-338.1 but had no effect on binding of aSyn-336.1. Isothermal titration calorimetry was conducted to determine the monovalent affinities of aSyn-323.1, aSyn-336.1 and aSyn-338.1 to synuclein peptides encompassing their respective epitope regions. Affinities of aSyn-323.1, aSyn-336.1 and aSyn-338.1 to their cognate peptides were 2.8 μM, 1.4 μM and 0.3 μM, respectively (Fig. S2), and binding was shown to be mediated by electrostatic interactions in all three cases (Fig. 2c).

**Fig. 3:**
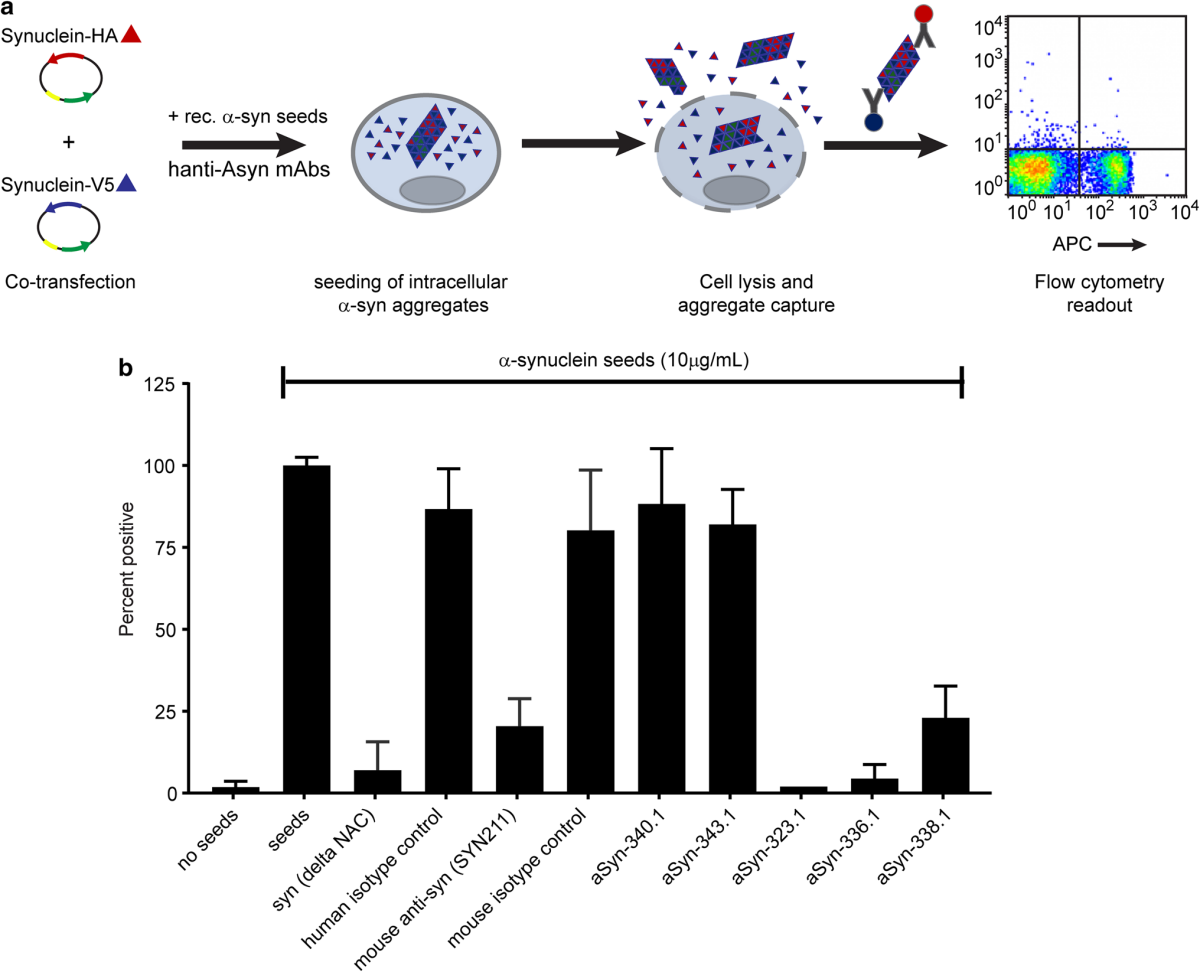
Functional activity of aSyn-323.1, aSyn-336.1 and aSyn-338.1. **a** Schematic representation of the in vitro synuclein seeding assay. Plasmids encoding synuclein with -V5 and -HA tags are co-transfected into HEK293 cells. The next day, a mixture of synuclein pre-formed fibrils (PFF) (serving as seeds) and anti-synuclein or control antibodies is added to the cells. 72 h later, cells are lysed and beads conjugated with anti-HA antibodies are used for immunoprecipitation. The formation of intracellular synuclein aggregates triggered by the seeds is detected by flow cytometry using anti-V5 antibodies labeled with APC as newly formed aggregates will contain both HA and V5 tags. Inhibition of synuclein seeding by the presence of anti-synuclein antibodies will result in reduced formation of aggregates, and thus a reduction in the percentage of APC-positive particles. **b** Percentage of APC-positive particles after incubation of cells without or with 10 μg/ml seeds, or with 10 μg/ml seeds and 500 μg/ml of the indicated antibodies normalized against that of samples incubated with seeds only. Samples were measured in quadruplicate. Error bars indicate SD

To assess whether binding of these antibodies may be able to interfere with the spreading of Lewy pathology, we established an in vitro α-synuclein seeding assay (Fig. 3a). Recombinant α-synuclein fibrils were formed in vitro and used as seeds in the assay. Mixing of the pre-formed α-synuclein fibrils with each of the three antibodies resulted in a dramatic decrease in the formation of intracellular synuclein aggregates (Fig. 3b). In contrast, mixing the seeds with antibodies aSyn-340.1 and aSyn-343.1 that were shown not to bind full-length α-synuclein (Fig. 2a) did not show any activity in the assay, as did a negative isotype control anti-RSV antibody.

**Fig. 4:**
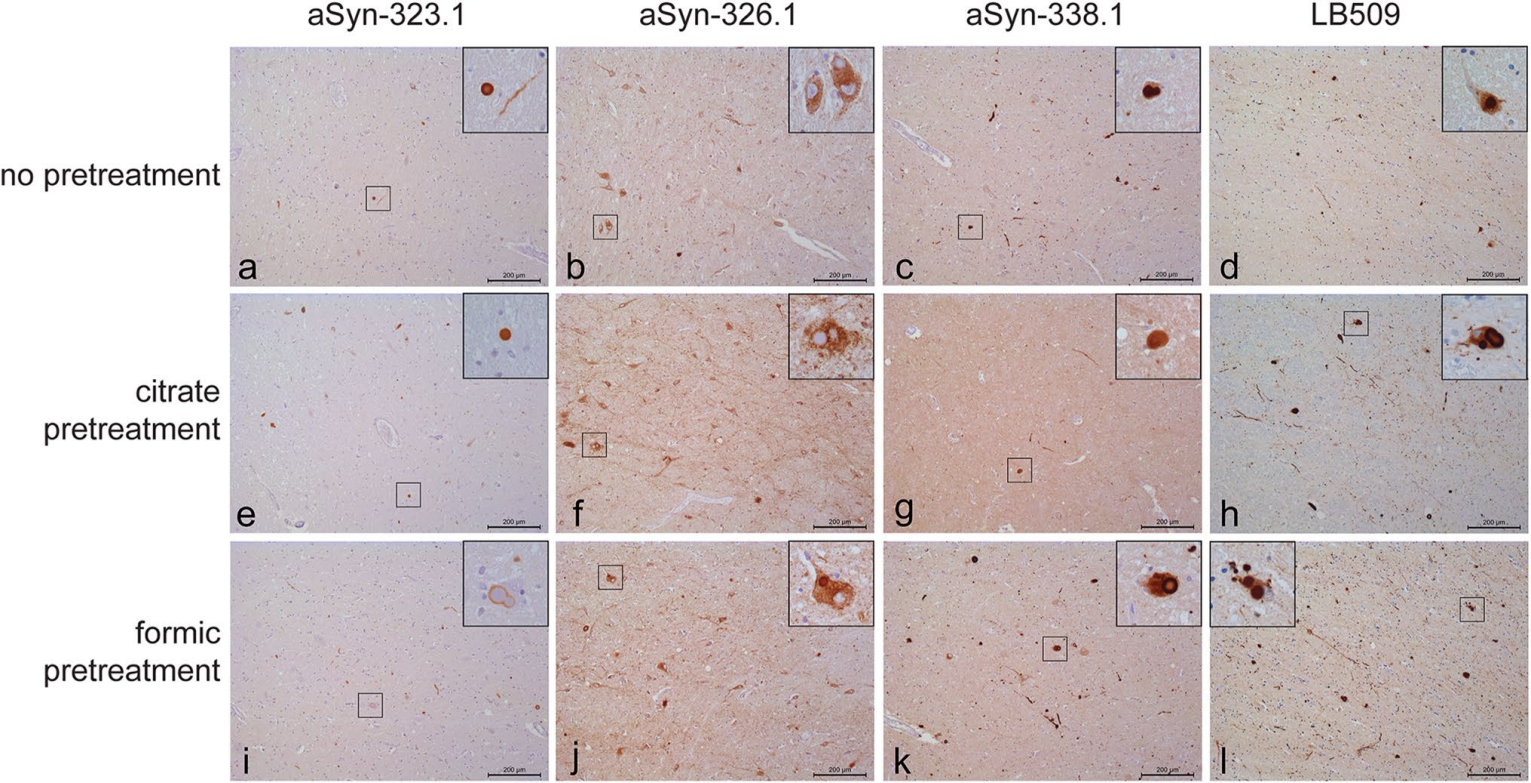
Immunohistochemical detection of pathological α-synuclein in PD brain tissue. Immunohistochemistry was performed on the mesencephalon of PD cases. **a**–**d** Immunostaining using antibodies aSyn-323.1, aSyn-336.1, aSyn-338.1 and LB509 without pre-treatment. **e**–**h** Immunostaining using antibodies aSyn-323.1, aSyn-336.1, aSyn-338.1 and LB509 after pre-treatment with citric acid. **i**–**l** Immunostaining using antibodies aSyn-323.1, aSyn-336.1, aSyn-338.1 and LB509 after pre-treatment with formic acid. Scale bar = 200 μm, for insets 50 μm

Finally, to assess whether human anti-α-synuclein antibodies aSyn-323.1, aSyn-336.1 and aSyn-338.1 recognize actual Lewy pathology, we conducted immunohistochemistry on post-mortem PD brain tissue. In the substantia nigra pars compacta (SNc), an area severely affected in PD, accumulation of α-synuclein can be detected in Lewy body (LB) and Lewy neurites (LN). Antibody LB509, detecting an epitope encompassing amino acids 115–122 of α-synuclein, was used as comparison for the intensity and levels of alpha-synuclein immunostaining. Because of the possibility that epitopes could be masked due to formalin fixation or protein aggregation, we additionally assessed the immunoreactivity of aSyn antibodies after antigen retrieval using either citric acid or formic acid. Without antigen retrieval, antibody aSyn-323.1 showed moderate immunostaining of LB and LN, aSyn-336.1 showed immunostaining of the cytoplasm in neurons but no LB or LN, and aSyn-338.1 showed clear detection of LB and LN (Fig. 4a–c) in the SNc. The detection by aSyn-338.1 was comparable to the detection of LB and LN using LB509 under similar conditions (Fig. 4d). After antigen retrieval using citric acid, aSyn-323.1 showed weak detection of LN, aSyn-336.1 showed cytoplasmic staining in neurons in the absence of LB and LN, and aSyn-338.1 showed moderate staining of LB and some LN (Fig. 4e–g). After citric acid pretreatment, LB509 showed stronger immunoreactivity and higher levels of LB and LN as compared to the other antibodies. After formic acid pretreatment, aSyn-323.1 showed weak reactivity, only detecting the outer rim of LB. aSyn-336.1 showed weak staining of LB, and aSyn-338.1 showed a clear detection of LB and LN comparable to the detection observed with LB509 (Fig. 4i–l). In summary, pretreatment of PD brain tissue samples resulted in immunoreactivity to pathological α-synuclein with aSyn-323.1, aSyn-336.1 and aSyn-338.1.

## Discussion

Definitive diagnosis for early stage of Parkinson’s disease is still difficult to achieve. Current diagnostic methods have minimal accuracy and can be expensive or invasive (reviewed in [9]). Therefore, there is a need for serum-based biomarkers for diagnosis of early stage PD. However, using serum levels of α-synuclein as a biomarker is still unconvincing [13] and, as described in the introduction, conflicting results have been reported for the difference in overall serum levels of auto-antibodies against α-synuclein between PD patients and healthy controls. Isolation and characterization of naturally occurring auto-antibodies can potentially lead to the identification of antibodies targeting specific (neo-)epitopes directly associated with pathology that can, therefore, serve as biomarkers. The recovery of α-synuclein auto-antibodies from the memory B-cell repertoire of PD patients described here establishes this compartment as a potential source of such discriminative antibodies.

Both passive and active immunization approaches targeting α-synuclein have been studied in mice [14, 25, 26, 37, 39]. Masliah et al. [25] showed that active immunization of human α-synuclein transgenic mice with recombinant human α-synuclein protein resulted in decreased accumulation of aggregated α-synuclein in neuronal cell bodies and synapses, and ameliorated neurodegeneration. Passive immunization with monoclonal antibodies against the C-terminus of α-synuclein was shown to ameliorate the behavioral deficits associated with α-synuclein deposition in mouse models of synucleinopathy [4, 14, 26]. In mice injected with synthetic α-synuclein fibrils, injection of an antibody against N-terminus of α-synuclein improved Lewy body pathology and reduced neurodegeneration [39]. Finally, clinical trials of therapies directly targeting α-synuclein include both active immunotherapy with vaccines PD01A and PD03A (AFFiRiS) [32] and passive immunotherapy with antibodies PRX002 [31] and BIIB054 [42].

Our efforts led to the recovery of 10 anti-α-synuclein antibodies from the memory B-cell repertoire of PD patients. The recovered antibodies were isotype switched and showed a degree of somatic mutation suggestive of an ongoing antigen-driven immune response. Of these ten antibodies, the three antibodies that showed the best binding to full-length, non-phosphorylated synuclein protein were characterized further. Antibodies aSyn-323.1, aSyn-336.1, and aSyn-338.1 inhibited the “seeding” of intracellular synuclein aggregates in an in vitro synuclein aggregation assay (Fig. 3). In addition, all three antibodies recognized Lewy pathology in post-mortem PD brain tissue samples (Fig. 4), albeit with differential reactivities across the tissue treatment conditions used. While pS129 and pS87 are important phospho-epitopes that are associated with LB pathology in PD [13, 27, 28], our results suggest that non-phospho-epitopes may also be relevant to disease as aSyn-323.1, aSyn-336.1, and aSyn-338.1 effectively inhibit aggregation and recognize LB pathology in PD tissue sections. Additional studies are needed to assess the toxicity and impact on cell viability from full-length α-synuclein in the in vitro aggregation model described here, and whether the α-synuclein antibodies can inhibit the toxicity of additional synuclein species, including previously reported non-fibrillar truncations [15].

The results described here suggest that the memory B-cell repertoire of PD patients might represent a potential source of α-synuclein antibodies, which may help to define disease-relevant epitopes that could be leveraged as biomarkers and/or therapies. While only three antibodies are described here, further studies are needed to better understand the breadth of anti-alpha-synuclein antibodies specificities that are not only present in PD patients, but likely present across the general population. A clear understanding of such specificities and their distinct properties is needed to assess their potential for use as biomarkers and/or therapy.

## Electronic supplementary material

Below is the link to the electronic supplementary material.

Supplementary material 1 (PDF 654 kb)
