# Supplementary material for: Naturally occurring antibodies isolated from PD patients inhibit synuclein seeding in vitro and recognize Lewy pathology
Source: Acta Neuropathol. 2019 Feb 25;137(5):825–36. doi: 10.1007/s00401-019-01974-5 (PMC6482120; doi:10.1007/s00401-019-01974-5)
Supplement: Supplementary file 1 — Supplementary material 1 (PDF 654 kb) [file 401_2019_1974_MOESM1_ESM.pdf]

**Supplementary Information**

# Naturally-occurring antibodies isolated from PD patients inhibit synuclein seeding *in vitro* and recognize Lewy pathology

Xinyi Li[1], Wouter Koudstaal[2,a], Lauren Fletcher[1], Martha Costa[1], Margot van Winsen[2], Berdien Siregar[2], Hanna Inganäs[2], Julie Kim[1], Elissa Keogh[1], Jeremy Macedo[1], Trevin Holland[1], Stuart Perry[1], Frederique Bard[1], Jeroen J. Hoozemans[3], Jaap Goudsmit[2,4,5], Adrian Apetri[2,b], Gabriel Pascual[1,b]

[1]Janssen Prevention Center, Janssen Pharmaceutical Companies of Johnson & Johnson, 3210 Merryfield Row, San Diego, CA 92121, USA. [2]Janssen Prevention Center, Janssen Pharmaceutical Companies of Johnson & Johnson, Archimedesweg 6, 2333 CN, Leiden, the Netherlands; [3]Department of Pathology, Amsterdam Neuroscience, VU University Medical Center, De Boelelaan 1117, 1081 HV, Amsterdam, the Netherlands; [4]Department of Epidemiology, Harvard T.H. Chan School of Public Health, 677 Huntington Avenue, Boston, MA 02115, USA; [5]Department of Neurology, Amsterdam Neuroscience, Academic Medical Center, Meidreefberg 9, 1105 AZ Amsterdam, the Netherlands.

[a]Present address: Lucidity Biomedical Consulting, Calle Emir 11, 18006, Granada, Spain

[b]To whom correspondence should be addressed: GPascual@its.jnj.com; AApetri@its.jnj.com

## Table S1: Peptides and protein used in the study

| | Peptide | Residues | Amino Acid sequence |
|---|---|---|---|
| | syn1-25 | Syn 1-25 | MDVFMKGLSKAKEGVVAAAEKTKQG |
| | syn18-44 | Syn 18-44 | AAEKTKQGVAEAAGKTKEGVLYVGSKT |
| | syn40-65 | Syn 40-65 | VGSKTKEGVVHGVATVAEKTKEQVTN |
| | s17 | Syn101-120 | GKNEEGAPQEGILEDMPVDP |
| | s18 | Syn111-130 | GILEDMPVDPDNEAYEMPSE |
| | s19 | Syn121-140 | DNEAYEMPSEEGYQDYEPEA |
| Bait | pS129-(111-140)-LCBiot | Syn 111-140 | GILEDMPVDPDNEAYEMP(pS)EEGYQDYEPEA-LCBiot |
| Bait | LCBiot-pS87-(73-93) | Syn 73-93 | LCBiot-GVTAVAQKTVEGAG(pS)IAAATG |
| Bait | 61-95-LCBiot | Syn 61-95 | EQVTNVGGAVVTGVTAVAQKTVEGAGSIAAATGFV-K-LCBiot |
| Bait | LCBiot-61-95 | Syn 61-95 | LCBiot-EQVTNVGGAVVTGVTAVAQKTVEGAGSIAAATGFVK |
| Bait | α-Synuclein-Biot | Syn 1-140 | Full Length-Biot |
| Bait | LCBiot-79-110 | Syn 79-110 | LCBiot- QKTVEGAGSIAAATGFVKKDQLGKNEEGAPQE |
| Bait | LCBiot-100-120 | Syn 100-120 | LCBiot-LGKNEEGAPQEGILEDMPVDP |
| Bait | 121-140-LCBiot | Syn 121-140 | DNEAYEMPSEEGYQDYEPEA-LCBiot |
| | NP129-(111-140)-LCBiot | Syn 111-140 | GILEDMPVDPDNEAYEMPSEEGYQDYEPEA-LCBiot |
| | LCBiot-NP87-(73-93) | Syn 73-93 | LCBiot-GVTAVAQKTVEGAGSIAAATG |
| | A6940 | Tau 42-103 | GLKESPLQTPTEDGSEEPGSETSDAKSTPTAEDVTAPLVDEGAPGKQAA AQPHTEIPEGTTA |
| | A7416 | Tau 166-211 | ANATRIPAKTPPAPKTPPSSGEPPKSGDRSGYSSPGSPGTPGSRSR |
| | NPAT8 | Tau 194-212 | RSGYSSPGSPGTPGSRSRT |
| | A6907 | Tau 186-253 | GEPPKSGDRSGYSSPGSPGTPGSRSRTPSLPTPPTREPKKVAVVRTPPKSP SSAKSRLQTAPVPMPDL |
| | A6897 | Tau 299-369 | HVPGGGSVQIVYKPVDLSKVTSKCGSLGNIHHKPGGGQVEVKSEKLDF KDRVQSKIGSLDNITHVPGGGNK |
| | A7024 | Tau 139-198 | DKKAKGADGKTKIATPRGAAPPGQKGQANATRIPAKTPPAPKTPPSSGE PPKSGDRSGYS |

SA, Streptavidin conjugated -APC or -PE

Bait are peptides used in BSelex screening.

Numbering for Tau peptides corresponds to the 2N4R tau isoform.

**Fig. S1** Recombinant full length α-Synuclein-Biot purification and quality analysis. **a** SDS-PAGE under non-reducing conditions of different fractions from preparative SEC. **b** Analytical SEC of final batch. c SEC-MALS binding experiment of biotinylated α-synuclein with streptavidin-PE. The highly pure fractions D4, 5, 6, 7, 8, 9 and 10 were pooled together as the final batch.

**Fig. S2** Affinity of aSyn-323.1, aSyn-336.1 and aSyn-338.1 to synuclein peptides. Isothermal Titration Calorimetry (ITC) measurements for aSyn-323.1 with peptide syn19 (Syn121-140) **a**, aSyn-336.1 with peptide syn18 (Syn111-130) **b,** and aSyn-338.1 with peptide syn19 (Syn121-140)**c**. Variation in enthalpy is observed following incremental addition of mAb stock to synuclein peptide. No binding of antibodies to negative control peptide syn17 (Syn101-120) was observed. Continuous lines represent the best fit of experimental data assuming a single set of binding sites. Experiments were performed in PBS. Equilibrium dissociation constants ($K_d$) are shown on the individual graphs. The amino acid sequences of synuclein peptides are shown in Table S1.

Fig. S1

a

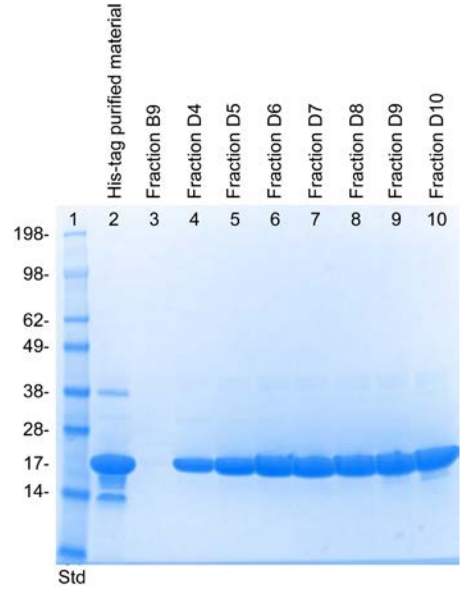

His-tag purified material
Fraction B9
Fraction D4
Fraction D5
Fraction D6
Fraction D7
Fraction D8
Fraction D9
Fraction D10
1 2 3 4 5 6 7 8 9 10
198-
98-
62-
49-
38-
28-
17-
14-
Std


b

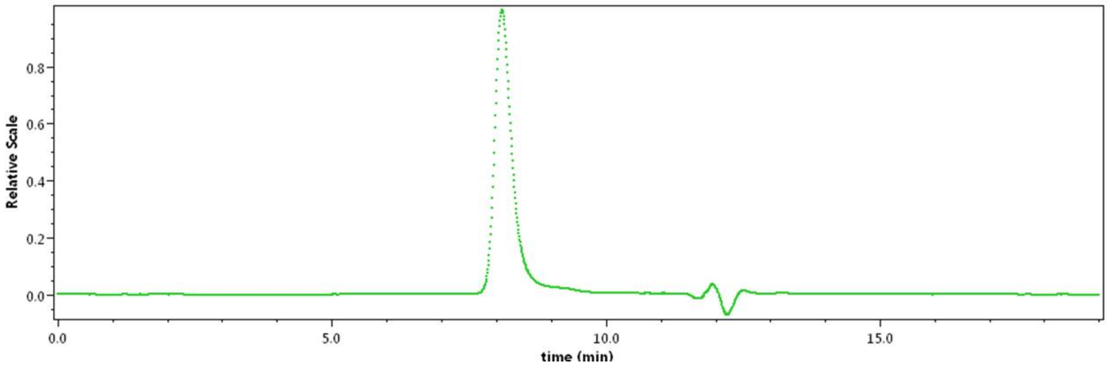

Relative Scale
0.0
0.2
0.4
0.6
0.8
0.0
5.0
10.0
15.0
time (min)


c

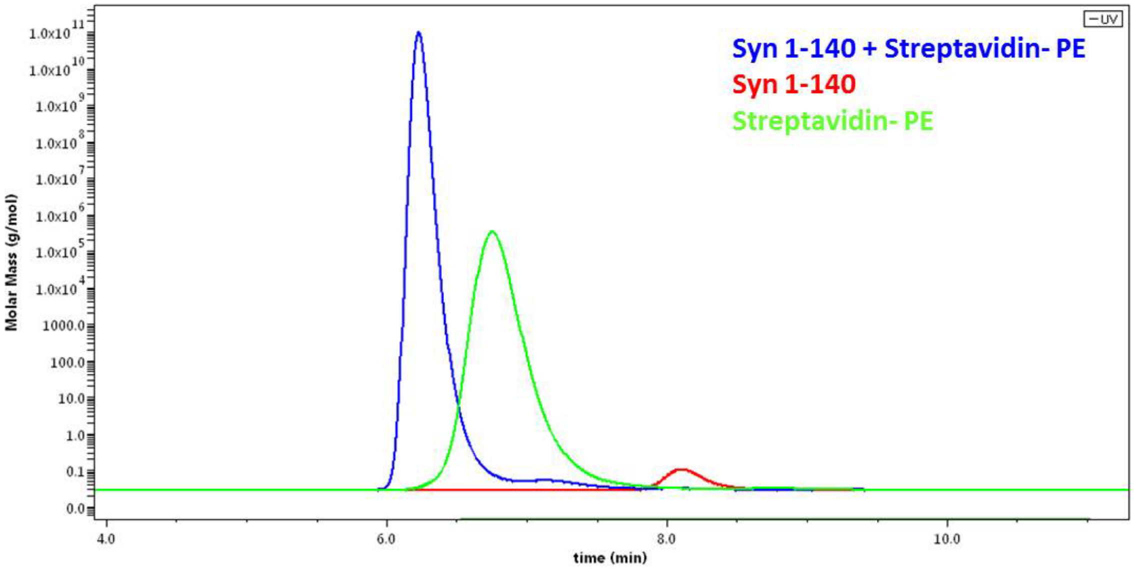

—UV
Syn 1-140 + Streptavidin- PE
Syn 1-140
Streptavidin- PE
Molar Mass (g/mol)
time (min)
4.0
6.0
8.0
10.0

Fig. S2

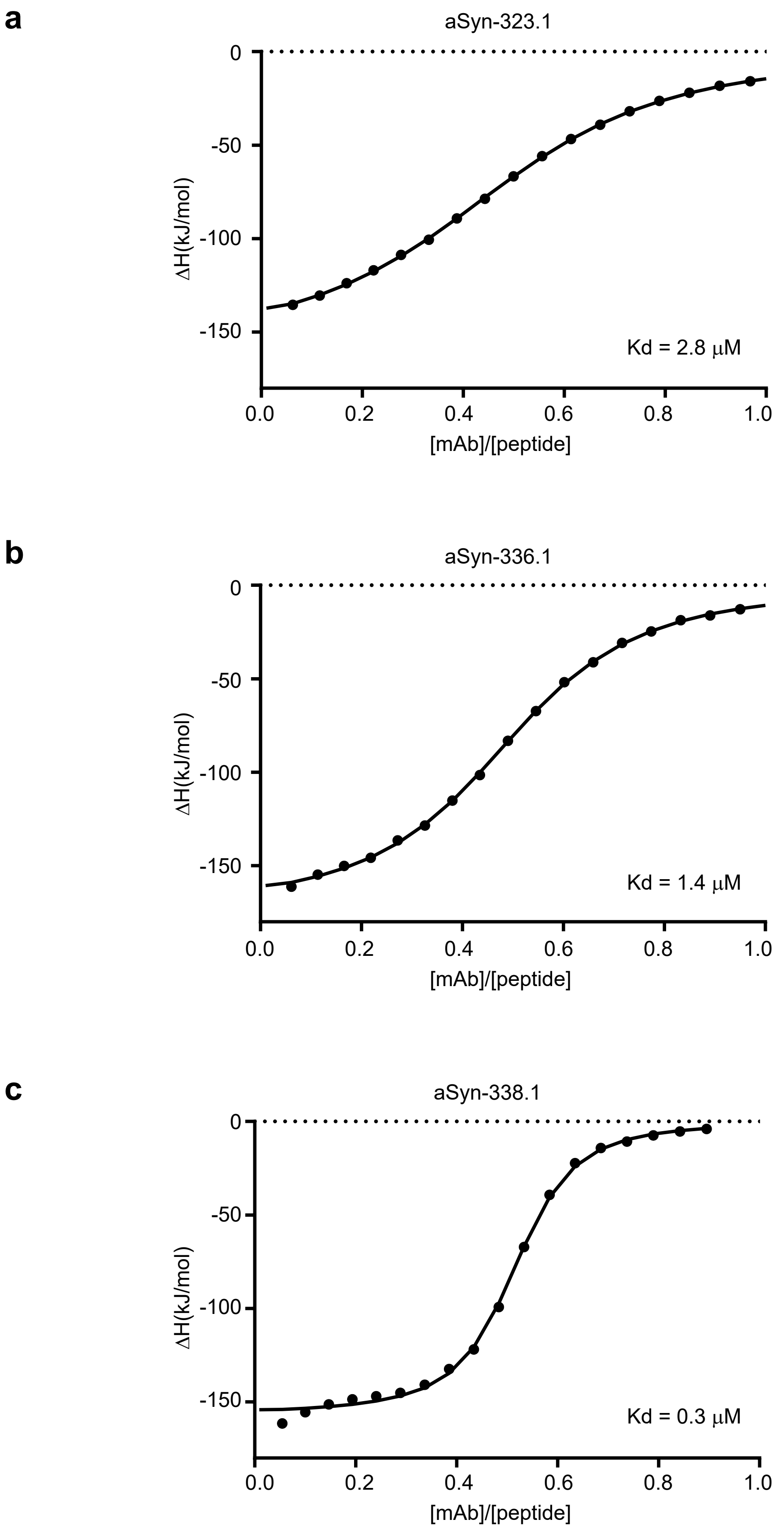
a
aSyn-323.1
0
-50
-100
-150
ΔH(kJ/mol)
Kd = 2.8 μM
0.0
0.2
0.4
0.6
0.8
1.0
[mAb]/[peptide]
b
aSyn-336.1
0
-50
-100
-150
ΔH(kJ/mol)
Kd = 1.4 μM
0.0
0.2
0.4
0.6
0.8
1.0
[mAb]/[peptide]
c
aSyn-338.1
0
-50
-100
-150
ΔH(kJ/mol)
Kd = 0.3 μM
0.0
0.2
0.4
0.6
0.8
1.0
[mAb]/[peptide]
